# On the Microstructure, Residual Stress and Fatigue Performance of Laser Metal Deposited TC17 Alloy Subjected to Laser Shock Peening

**DOI:** 10.3390/ma15186501

**Published:** 2022-09-19

**Authors:** Zhibin An, Weifeng He, Xin Zhou, Liucheng Zhou, Xiangfan Nie

**Affiliations:** 1 Science and Technology on Plasma Dynamic Laboratory, Air Force Engineering University, Xi’an 710038, China; 2 Institute of Aeronautics Engine, School of Mechanical Engineering, Xi’an Jiaotong University, Xi’an 710038, China

**Keywords:** laser melting deposition, laser shock peening, microstructure, fatigue

## Abstract

Laser shock peening (LSP) has been employed to improve the mechanical properties of repaired aerospace engine components via laser metal deposition (LMD). This study looked at cross-sectional residual stress, microstructure and high cyclic fatigue performance. The outcomes demonstrated that a compressive residual stress layer with a value of 240 MPa was formed at a depth of 200 μm in the laser melting deposited zone and the microhardness was improved by 13.1%. The findings of electron backscatter diffraction (EBSD) and transmission electron microscopy (TEM) analysis revealed that misorientation increased and dislocation features were observed after LSP which is beneficial to the enhancement of fatigue performance. The high cycle fatigue data illustrated that the LMD+LSPned samples exhibited 61% improvement in comparison to the as-LMD samples. In the aerospace sector, LSP and LMD are therefore very effective and promising techniques for restoring high-value components.

## 1. Introduction

TC17 (Ti-5Al-2Sn-2Zr-4Mo-4Cr) titanium alloy has been widely employed in manufacturing aerospace engine components such as compressor blisks due to its outstanding mechanical strength, excellent fracture toughness, high fatigue strength and good thermal stability [1]. It was initially developed by the U.S. Air Force and General Electric Company for manufacturing aircraft engine compressor disks. Nowadays, TC17 alloy is used to manufacture aero-engine blades, compressor disks, blisks and heavy section forgings for gas turbine engine components. However, due to harsh environments (e.g., high pressure, temperature, foreign body damage (FOD)), aero-engine components are often cracked during service time. The structural complexity of these components determine that remanufacturing the damaged ones is financially rewarding instead of replacing them with newer ones. Therefore, the advanced repair technology of titanium alloys plays an important role to reduce engine manufacturing time and maintenance costs [2].

Laser metal deposition (LMD) is an advanced near-net-shape additive manufacturing technique in manufacturing or repairing 3D components by adding materials layer-by-layer via a CAD model. LMD possesses various advantages including low cost, high flexible processing, short machining time, difficult machining materials and high material utilization [1]. Additionally, due to the rapid cooling rate during the LMD process, the LMD manufactured titanium possesses comparable or ever superior mechanical properties than that of the wrought titanium alloys [3]. In order to decrease the economic losses and the delivery time, LMD has been recently employed to repair high-value engine components. Zhu et.al. [4] investigated the tensile mechanical properties of laser additive manufactured TC11 titanium. The results illustrated that additively manufactured TC11 possesses good tensile strength of 1033 ± 13 MPa and elongation of 6.8 ± 0.2%. Anand Kumar et al. [5] have successfully repaired a nickel-based single crystal fabricated aero-engine blade with direct metal deposition. Additionally, previous investigations by Liu et al. [1] have demonstrated that the tensile strength and elongation of the laser additively repaired TC17 could reach 1100 MPa and 10%, accounting for 91–98% of that of wrought TC17 alloy. Kun et al. [6] employed point-mode forging and laser metal deposition to repair the Ti6Al4V alloy. The 50% repaired-volume Ti6Al4V alloy exhibited better performance on yield strength, ultimate tensile strength and elongation than the ASTM B381-2013 standard.

Although LMD could recover the original geometry morphology of the damaged components with certain recovered mechanical properties, components repaired by LMD still have issues such as tensile residual stress (TRS; formed during the LMD), reduced fatigue strength, etc. Therefore, another technique called laser shock peening (LSP) was employed to eliminate the tensile residual stress and improve the fatigue lives of LMD repaired components. LSP is an effective surface strengthening modification technique that can refine the surface microstructure, optimize the surface topology and induce compressive residual stress, thereby extending the fatigue life [7]. Therefore, researchers started to employ LSP as a post-strengthening processing technique in combination with laser additive manufacturing to improve the properties of the as-repaired components. Tong et al. [8] strengthened laser additively manufactured CoCrFeMnNi high-entropy alloy with a Q-switched Nd-YAG LSP system. Nanoscale grains were formed on the surface layer due to severe plastic deformation by LSP. Similar investigations on the repairing technique were also carried out on the Ti6Al4V titanium alloy. Guo et al. [9] turned the tensile residual stress on the additively manufactured Ti6Al4V samples to compressive residual stress with an affected depth of around 700 µm. Lu et al. [10] printed Ti6Al4V via a selective laser melting technique, followed by LSP. The tensile results show that the ultimate tensile strength of SLMed samples is of 1004 MPa and 997 MPa while those of SLM-LSPned samples are 1287 MPa and 1197 MPa in two printing directions. The high strength and ductility can be obtained in SLM+LSPned samples due to the formation of nanomechanical twins by LSP, leading to improvements of 14.3% and 18.3% in Lv et al.’s work [11]. LSP can also be combined with wire and arc additive manufacture (WAAM) [12] and the WAAM+LSPned samples exhibited higher elongation after LSP. Additionally, Guo et al. [13] examined the high-temperature oxidation resistance of additive manufactured TC4 subjected to LSP. It was found that after LSP, the Al-rich layer was changed to three layers that effectively prevent the diffusion of oxygen, thereby strengthening the oxidation resistance of the TC4 alloy.

Our previous study was conducted in the cycle of 10^5^ and a fatigue experiment was carried out on the strengthened and repaired TC17 aero-engine blades by the combination techniques of LMD and LSP [14]. In this work, we firstly investigated the effects of LSP on the high-cycle fatigue performance (>10^6^ cycles) of an as-LMDed TC17 alloy before and after LSP. Residual stress and microhardness were measured via the hole drilling method and Viker’s indentation method. The microstructure was characterized by EBSD and TEM to investigate the effect of LSP on the microstructure of laser additively manufactured samples. Finally, the combination technique was a promising method to apply to the repairing of aero-engine blades.

## 2. Experiment and Microstructure Characterization

### 2.1. Materials

TC17 (Ti-5Al-2Sn-2Zr-4Mo-Cr) powder was supplied by Haibao Ltd., Hunan, China. The elemental contents are given in Table 1. The diameter of spherical particles varied from 60 µm to 140 µm, measured using a laser diffraction particle analyzer.

### 2.2. Laser Melting Deposition and Laser Shock Peening

**Figure 1:**
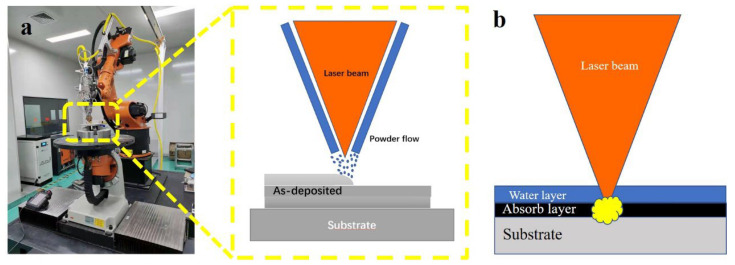
The schematic image of LMD (**a**) and LSP (**b**).

**Figure 2:**
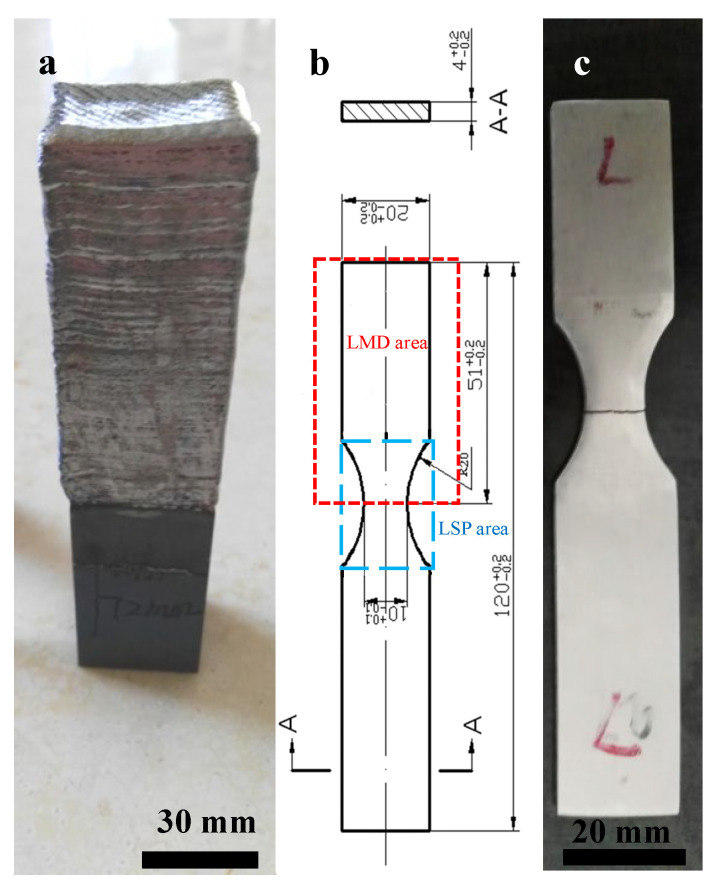
The laser repaired sample (**a**), the dimensions of the fatigue sample with LSP and LMD areas (**b**) and the fractured as-LMD sample (**c**).

As shown in Figure 1, the schematic image of LMD (a) and LSP (b), a 4 kW fiber laser with a laser wavelength of 1064 nm, a nozzle with a four-way coaxial powder feeder and an ABB six-axis robot were employed to carry out the LMD experiment. The detailed LMD processing parameters are given in Table 2. A TC17 plate with dimensions of 100 mm by 100 mm by 30 mm was used as substrate. The laser melting deposited fatigue samples were designed as 130 mm by 30 mm by 50 mm (as shown in Figure 2a) and machined to the dimensions shown in Figure 2b,c. The LSP process area is in the center of the fatigue sample. Both sides of the fatigue sample were strengthened by LSP.

An LSP schematic diagram is given in Figure 1a. A nanosecond pulsed Nd: YAG laser system manufactured by Tyrida, Ltd. was employed. The calculated laser density for each pulse was 3.5 GW/cm^2^. The wavelength is 1064 nm, the pulse duration is 15 ns. Black tape was used as an absorbing layer and tap water (thickness of 1 mm) for confining plasma shock waves. The detailed LSP processing parameters are listed in Table 2.

### 2.3. Microstructure Characterization and Mechanical Property Measurement

**Figure 3:**
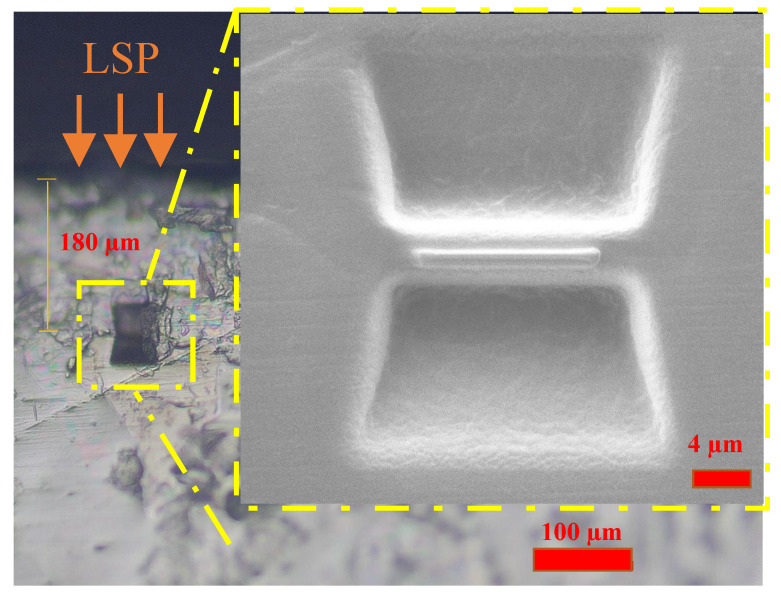
The position of the TEM lamella sample using the in situ lift-out FIB method.

The microstructure of additively manufactured alloy was characterized by SEM/EDS, EBSD and TEM techniques. The as-received bulk sample with dimensions of 5 mm by 5 mm by 5 mm was cut into sample pieces, followed by the procedures of grinding using silicon carbide papers and polishing with 9 µm, 3 µm and oxide polishing suspensions (OPSs) to a mirror-like surface. The EBSD detector (symmetry, Oxford instrument, Oxford UK) was equipped with a field emission scanning electron microscope (FESM, JEOL Model 7800, Tokyo, Japan). The texture was characterized by orientation density function (ODF) and the deviation angle for the texture component was 20°. The TEM sample was prepared with a focusing ion beam (FIB) on a Zeiss SEM 440 Model using various currents (30 nA, 3 nA, 300 pA, 50 pA and low kV polishing), as shown in Figure 3. A JEOL 2100F electron microscope was used for TEM observation.

The vibration fatigue test was conducted with a QBG-100 fatigue tester (Jinan, China) at room temperature. All samples were tested with tension–tension (axial) fatigue at a 20 Hz frequency and an R = 0.1 stress ratio, and the applied stress was 300 MPa. For each condition, the fatigue tests were repeated three times for statistical purposes. In terms of residual stress measurement, detailed procedures can be found in [15]. Finally, a SEM was employed to analyze the fracture morphologies of the fractured samples.

## 3. Results

### 3.1. Microhardness and Residual Stress

**Figure 4:**
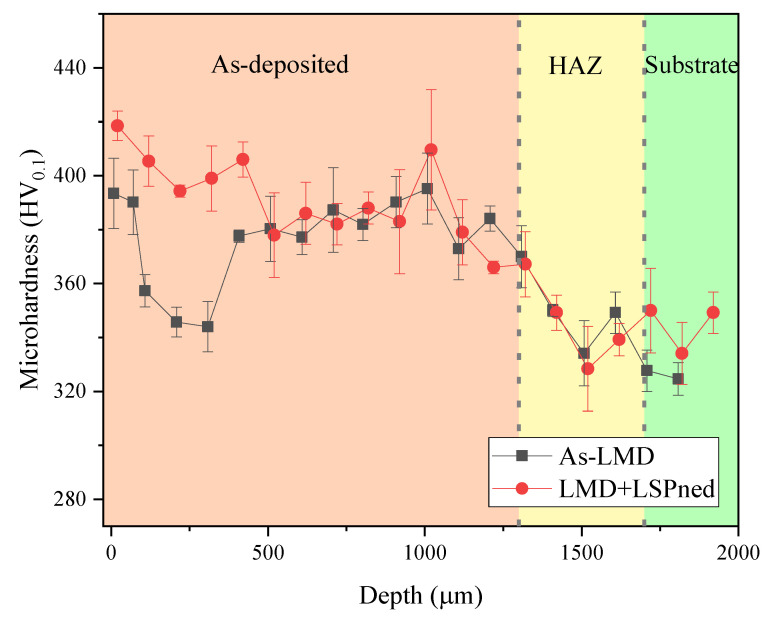
The cross-sectional microhardness distribution of as-LMD and LAM+LSPned samples.

The higher surface hardness exhibited better performance on wear resistance and foreign object damage tolerance [16,17]. Figure 4 illustrates the cross-sectional microhardness distribution of as-LMD and LMD+LSPned samples. It can be seen that the microhardness of the as-deposited area is harder than the heat-affected zone and substrate. The microhardness in the substrate area is around 320 HV_0.1_ while the surface microhardness of the as-LMD sample is around 380 HV_0.1_. After LSP, this value is increased to 430 HV_0.1_ with an increase of 13.1%.

**Figure 5:**
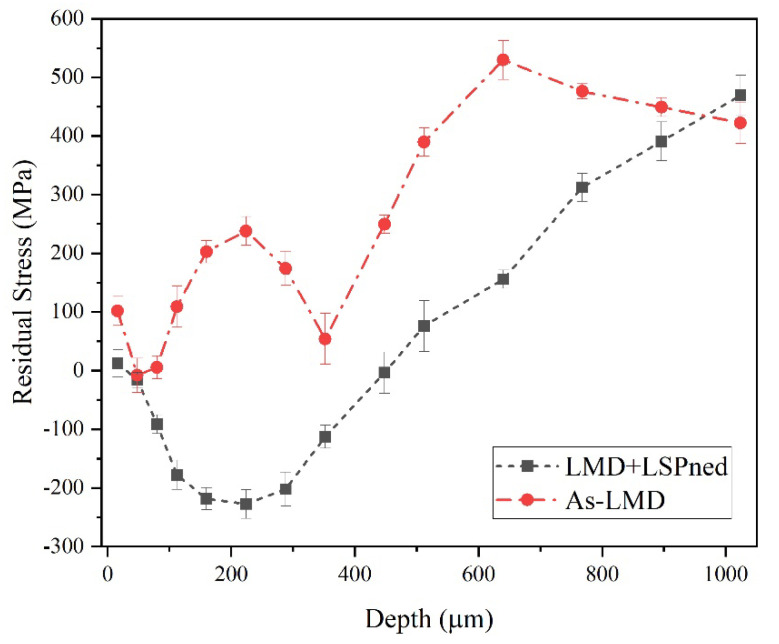
The in−depth residual stress distribution of as-LMD and LAM+LSPned samples.

With the effect of the laser-induced plasma shock waves, compressive residual stress was formed in the material along the cross-sectional direction [15,18]. Figure 5 shows the in-depth residual stress curves of as-LMD and LMD+LSPned samples. The in-depth surface of as-LMD samples exhibited an non-uniform tensile stress layer from the top surface of 100 MPa then fluctuated to its top peak of 500 MPa at the depth of 600 MPa. By contrast, after LSP, the tensile stress was transferred into compressive residual stress. The compressive residual stress reaches the maximum (−240 MPa) at around 200 µm. Then, the compressive residual stress decreases to 0 MPa at the depth of 500 µm and as the depth increases, the compressive residual stress transfers to tensile status. This is because the shock waves decline along the in-depth direction and less plastic deformation is generated [19].

### 3.2. Microstructure Characterization

**Figure 6:**
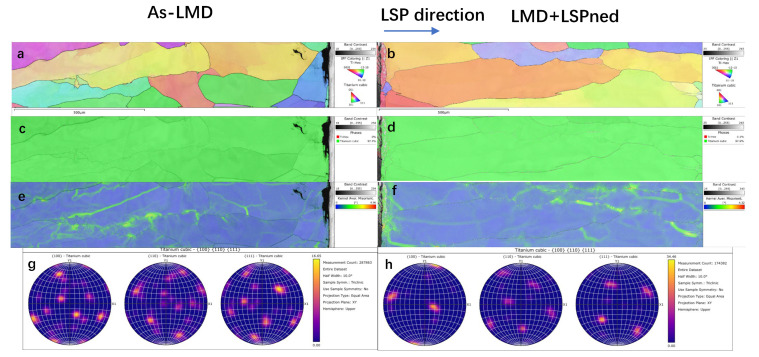
EBSD analysis of as-deposited area of as-LMD and LMD+LSPned samples: (**a**,**b**) IPF Z mappings; (**c**,**d**) phase mappings; (**e**,**f**) KAM mappings; (**g**,**h**) pole figures.

**Figure 7:**
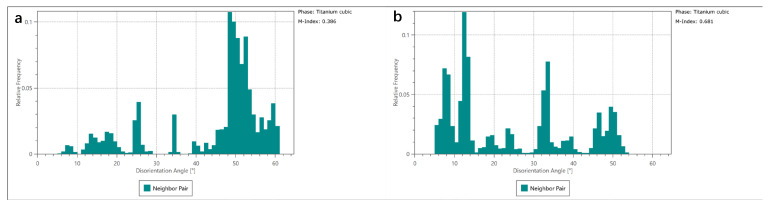
The corresponding misorientation statistics of cubic titanium in the as-deposited area. (**a**) as-LMD, (**b**) LMD+LSPned.

Figure 6 illustrates the EBSD mappings of the as-deposited area of as-LMD and LMD+LSPned samples. According to the IPF Z and pole figures (as shown in Figure 6a,b), the grain orientation of the as-deposited area is distributed randomly. The phase mappings (as shown in Figure 6c,d) show the as-deposited area consisting of cubic titanium and there is no hcp-Ti formed. This corresponded with the work by [4]. Misorientation is the difference in crystallographic orientation between two crystallites in a polycrystalline material. By comparing the misorientation of as-LMD and LMD+LSPned samples, as illustrated by the misorientation angles in Figure 6e,f and Figure 7, it can be seen that after LSP the misorientation index was increased from 0.386 to 0.681. LSP can greatly increase the misorientation in the as-LMD sample.

**Figure 8:**
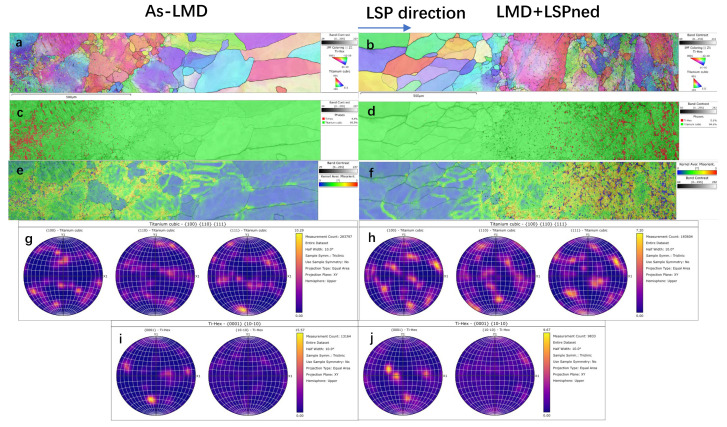
EBSD analysis of HAZ area of as-LMD and LMD+LSPned samples. (**a**,**b**) IPF Z mappings; (**c**,**d**) phase mappings; (**e**,**f**) KAM mappings; (**g**,**h**) pole figures for hcp and cubic phase. (**i**,**j**) pole figures for cubic phase.

**Figure 9:**
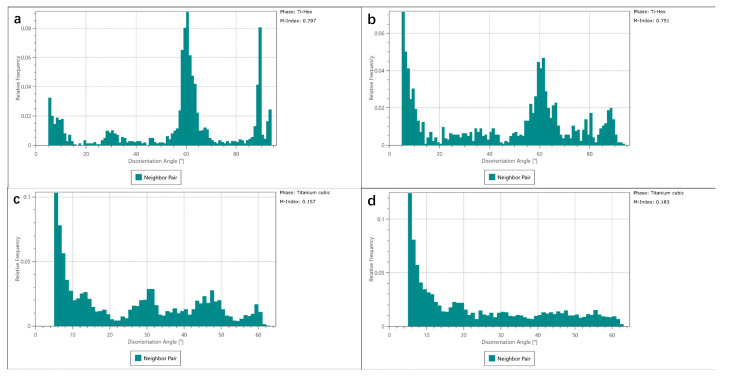
The corresponding misorientation statistics of cubic titanium and hcp titanium of as-LMD (**a**,**c**) and LMD+LSPned samples (**b**,**d**) in the HAZ area.

Additionally, Figure 8 shows the IPF Z, KAM and phase mappings in the heat affected zone (HAZ). In this area, as illustrated by Figure 8c,d, Ti-Hex phase starts to emerge in the HAZ area and the matrix phase is the Ti-cubic phase. The grain orientations of cubic and hcp titanium are still random as shown in Figure 8a,b and Figure 8g–j. Unlike the as-deposited area, the effects of LSP on the misorientation are limited as illustrated by KAM maps in Figure 8e,f and the misorientation index in Figure 9. In both as-LMD and LMD+LSPned samples, the misorientations are much higher in the HAZ area than that in the as-deposited area. Additionally, the misorientation index values of cubic and hcp titanium of as-LMD samples are 0.157 and 0.797 while such values of LMD-LSPned samples are 0.183 and 0.751, respectively. There are nearly no changes in the misorientation between as-LMD and LMD+LSPned samples. This is because the LSP-induced microstructure movements cannot reach the inner HAZ.

**Figure 10:**
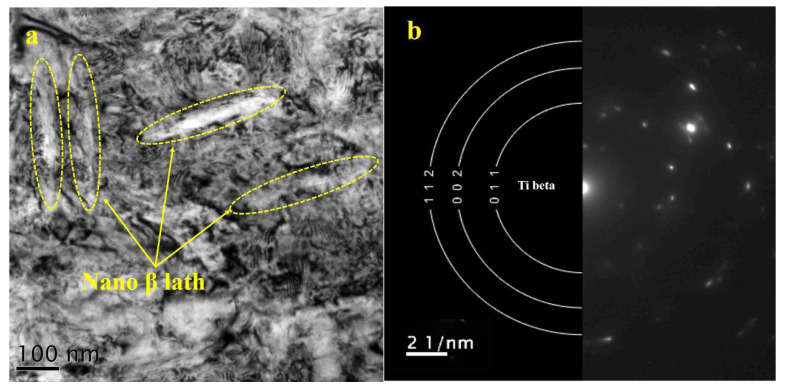
TEM image of the as-deposited area (**a**) and its corresponding SEAD image (**b**) of the LMD+LSPned sample.

It is well known that LPS-induced strain on the surface can reach a 10^6^/s level and the plastic strain decays from the top surface to the inner microstructure [20]. As mentioned in the residual stress section and the as-deposited KAM results, it is necessary to further investigate the microstructure at the sub-surface of the LMD+LSPned sample. Therefore, TEM observations at the depth of around 180 µm were carried out. Figure 10 shows the TEM bright field image and the corresponding SEAD image of the LMD+LSPned sample at 180 µm. It can be seen that there are nanowide laths embedding in the matrix and, as indexed by SEAD, it is a beta lath. Since the size of the cubic lath is around 100 nanometers, it is impossible for it to be directly mapped by the EBSD technique. That is why in the EBSD data the grain size of the as-deposited results is very large. According to the Hall–Petch relationship (grain refinement strengthening mechanism), grain refining causes more grains in the affected region to be rotated to have various orientations and more grain barriers to form, which prevents dislocations from passing through, moving through and collecting between grains, slowing the spread of cracks and enhancing material working hardening. That is why, although according to the EBSD data it is coarse grain in the as-deposited area and fine grains in the substrate area, the microhardness of the as-deposited area is higher than that of the substrate area.

### 3.3. High Cycle Fatigue and Fractural Morphology Observation

**Figure 11:**
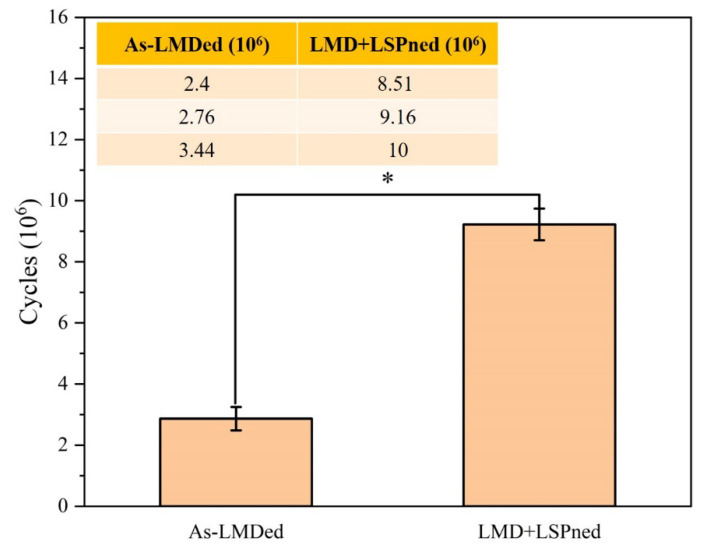
The vibration fatigue cycles of as-LMD and LMD+LSPned samples. (* *p* < 0.05 compared to the as-LMD).

The high cycle fatigue performance of as-LMD and LMD+LSPned samples is shown in Figure 11. The average fatigue cycle of as-LMD samples is around 2.867 × 10^6^ cycles while that of the LMD+LSPned samples is 9.22 × 10^6^ cycles. The fatigue cycles are counted automatically with three averages and the errors are in a small range that can be accepted. It can be seen that the fatigue cycles of as-LMDed samples vary from 2.4 to 3.44 (×10^6^) while those of LMD+LSPned vary from 8.51 to 10 (×10^6^). The fatigue performance is determined by both LMD and LSP. As we know, there are defects such as voids of unmelted powders and microcracks in the as-LMD sample that could dramatically influence the fatigue performance. Even with the defective sample, it can still be seen that there is a three-fold increase in the high cycle fatigue performance after laser shock peening, therefore LSP is an effective way to improve the fatigue performance of the LMD repaired components. However, it should also be noted that LSP has limited or even no effectiveness on defective repaired samples as indicated in [21]. Therefore, the repaired quality of the metallic sample is also one of the crucial factors in the fatigue performance.

In order to better understand the failure mechanism, the fractural morphologies were observed via SEM.

**Figure 12:**
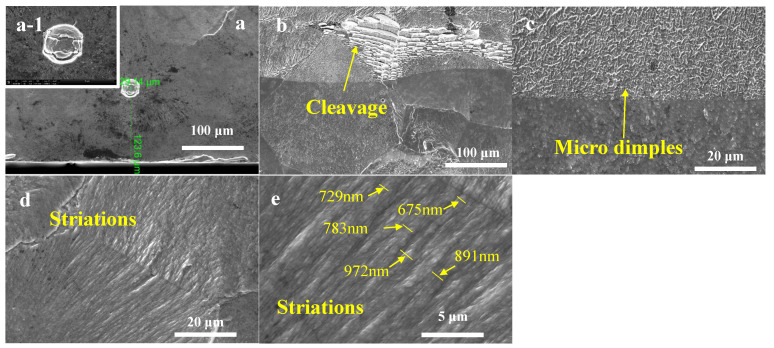
The facture morphologies of LMD+LSPned samples: (**a**) crack initial place; (**b**) cleavage facets; (**c**) microdimples; (**d**,**e**) fatigue striations.

The fracture surface was characterized by river-like patterns in LMD+LSPned samples, as shown in Figure 12. It was observed that the crack initiation sites at the sub-surface where the initiation sites were located at the depth of 120 µm. Figure 12a–e show typical fracture morphologies of the LMD-LSPned sample. The fracture morphology features smooth cleavage planes, illustrating a typical cleavage fracture mode in the LMD-LSPned sample as shown in Figure 12b. Additionally, microdimples and fatigue striations are also observed in Figure 12d,e, indicating ductile fracture also exists. Fatigue striations are helpful for limiting fatigue crack propagation [16]. According to previous work [19], a high density of fatigue striations suggests a slower rate of crack propagation. Additionally, the secondary cracks can also aid in slowing down the initial microcrack growth rates since they can consume lots of energy [22]. The width of the fatigue striations varies from 600 nm to 972 nm. Therefore, it can be seen that there is a mixture of ductile and cleavage fractures in the LMD+LSPned TC17 sample. Chi et al. [23] also observed a combination of morphologies of ductile and cleavage fractures in a DED+LSPned titanium alloy.

## 4. Discussion

In terms of the combination technique of laser shock peening and additive manufacturing, some researchers have investigated different materials and AM techniques and the results vary. Chi et al. [23] strengthened the direct energy deposited TA15/TC17 wall with laser shock peening. The UTS and YS of as-deposited TA15/TC17 were improved by 12.45% and 11.92% after LSP. Additionally, Lu et al. [24] also printed Ti6Al4V alloy by laser directed energy deposition (LDED), followed by LSP processing. The tensile results illustrate that UTS and elongation were increased by 20.8% and 67.2%. However, fatigue tests were not conducted on the LDED+LSPned samples. Jiang et al. [21] employed LSP to post-process the selective laser melted Ti6Al4V alloy and examined the ultra-high cycle fatigue performance. However, the fatigue results of the SLM+LSPned samples exhibited a lower S-N curve than as-SLMed samples. This is because the inherent defects such as unmelted powders and lack of fusion facilitate crack initiation, expansion and, finally, premature failure, thereby dominating the fatigue failure of the SLMed samples.

Its well known that the inherent TRS can deteriorate the mechanical properties of the metallic component. As discussed in Section 3.1, the inherent tensile residual stress was formed in the as-LMD sample as shown in Figure 5. The TRS increases the effective net stress range and the mean stress during fatigue loading, thereby accelerating the fatigue crack initiation and increasing the fatigue crack propagation rate.

**Figure 13:**
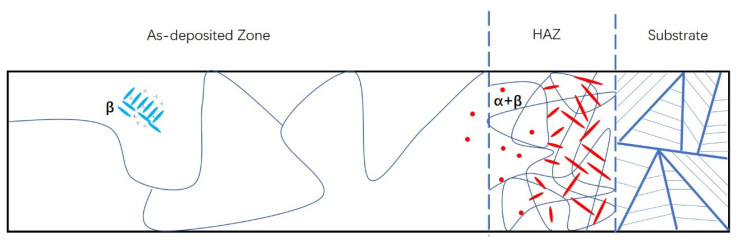
Schematic diagram showing the microstructure evolution in the LMD+LSPned sample.

Additionally, the inherent defects aided in early fracture initiation and propagation. As a result, the majority of fatigue cracks are initiated from inherent defects. The schematic microstructure of the LMD+LSPned sample is shown in Figure 13 which is consistent with that found by Zhu et al. [4]. As depicted in the substrate, the microstructure consists of lamellar α and β grains. In the HAZ, the α phase exists as a form of equiaxed grain distributed randomly in the β matrix. In the as-deposited area, the large β grains are observed with the EBSD technique, and there are nano β lath grains inside the large β grains as shown in the TEM image. The dislocations can be observed near the nano β lath grains as illustrated in Figure 13.

The greatest difference between as-LMD and LMD+LSPned samples is the in-depth residual stress field. There are micropores or voids in the sub-surfaces of both samples. These flaws are the result of gas entraining and rapid cooling rate brought by turbulence in the molten pool, which eventually results in stress concentration and the increase in the rate of crack growth under the fatigue load [11]. LSP transformed the residual stress in the surface of the as-LMD sample from the tensile to the compressive state. By contrast, during the crack growth process, compressive residual stresses reduce the effective applied stress and stress intensity factor range at the crack tip, simultaneously causing a crack closure effect, which decreases the crack propagation rate and consequently increases the cyclic life of the LMD+LSPned sample [25].

## 5. Conclusions

In this work, the fatigue performance of laser metal deposited TC17 samples before and after LSP was investigated. The main findings are as follows:

1. The tensile residual stress in the surface of as-LMD samples was transferred to compressive residual stress after LSP. The maximum CRS of 240 MPa was obtained at the depth of 200 µm.
2. The fatigue cycles of LMD+LSPned samples were increased by 212% compared to those of the as-LMD samples.
3. The fatigue cracks initiated from the defects formed during the 3D printing process and LSP-induced CRS can effectively delay the crack propagation, thereby increasing the fatigue lives of laser additive manufactured components.

## Figures and Tables

**Table 1:** Element composition of as-received TC17 powder.

Element	Al	Sn	Zr	Mo	Cr	H	O	N	Ti
Content wt%	4.6–5.4	1.6–2.6	1.5–2.5	3.6–4.4	3.5–4.6	<0.011	0.078–0.14	<0.05	Bal

**Table 2:** The detailed laser melting deposition and laser shock peening parameters.

LMD		LSP	
Laser Power	700 w	Laser Energy	5 J
Scanning Speed	500 mm/min	Spot Size	3 mm
Powder Flow	0.4 r/min	Overlap	50%
Spot Size	1.6 mm	Pulse Duration	20 ns
Overlapping	50%	Confinement Layer	water
